# Protein kinases as switches for the function of upstream stimulatory factors: implications for tissue injury and cancer

**DOI:** 10.3389/fphar.2015.00003

**Published:** 2015-02-18

**Authors:** Tina Horbach, Claudia Götz, Thomas Kietzmann, Elitsa Y. Dimova

**Affiliations:** ^1^Faculty of Biochemistry and Molecular Medicine, Biocenter Oulu, University of Oulu, Oulu, Finland; ^2^Department of Chemistry, University of Kaiserslautern, Kaiserslautern, Germany; ^3^Medical Biochemistry and Molecular Biology, Saarland University, Homburg, Germany

**Keywords:** USF, phosphorylation, transcription factor, cancer, tumor, signaling, therapeutic target

## Abstract

The upstream stimulatory factors (USFs) are regulators of important cellular processes. Both USF1 and USF2 are supposed to have major roles in metabolism, tissue protection and tumor development. However, the knowledge about the mechanisms that control the function of USFs, in particular in tissue protection and cancer, is limited. Phosphorylation is a versatile tool to regulate protein functions. Thereby, phosphorylation can positively or negatively affect different aspects of transcription factor function including protein stability, protein–protein interaction, cellular localization, or DNA binding. The present review aims to summarize the current knowledge about the regulation of USFs by direct phosphorylation and the consequences for USF functions in tissue protection and cancer.

## INTRODUCTION

All cells of an organism carry the same genetic information in their nucleic DNA. But the requirements of distinct cell types are different and they also can change upon environmental stimuli. Thus, the expression of genes into proteins has to be adjusted to the particular needs of a cell. This mainly occurs by transcriptional regulation of gene expression, and transcription factors play a pivotal role in this regard. Deregulated transcription factors are involved in the development of many severe diseases, and thus, elucidation of transcription factor regulation can provide valuable information regarding the development and treatment of these diseases.

Transcription factors are proteins that regulate gene expression by binding to the DNA sequences of their target genes and that can be classified according to their DNA-binding domain (DBD). Among the most important ones are homeodomain proteins, zinc-finger proteins, basic helix-loop-helix (bHLH) and leucine zipper (LZ) proteins. Often specific transcription factors contain ligand binding domains as well as one or more transactivating domains (TADs) permitting their ability to act as activators or repressors of gene expression.

With respect to the substantial role of transcription factors in gene expression regulation, it is not surprising that mutant transcription factors as well as deregulated transcription factors can cause tissue damage and thus severe diseases. In cancer, the inappropriate growth of cells is often caused by mutated or malregulated oncogenes or tumor suppressor genes, which, in turn, often encode transcription factors. Prominent examples are the oncogenic transcription factor c-Myc (Adhikary and Eilers, 2005) or the tumor suppressor p53, which is mutated in a variety of human cancers (Lane, 1992; Latchman, 2008).

The eukaryotic cell features several different mechanisms for the regulation of transcription factor function. One possibility is to tightly control the rate of transcription factor synthesis and/or degradation via the ubiquitin–proteasome system (Desterro et al., 2000). Posttranslational modifications are also a valuable tool in cellular transcription factor regulation. Beside SUMOylation (Verger et al., 2003; Gill, 2005) or acetylation (Bannister and Miska, 2000), protein phosphorylation plays a major role in this respect. In response to diverse extracellular signals, transcription factors can be phosphorylated by specific protein kinases or dephosphorylated by protein phosphatases. Protein phosphorylation is a dynamic process and the phosphorylation state of the transcription factor can affect its function in several ways: by changing the cellular localization, by regulating DNA binding and/or oligomerization of the transcription factor, by modulating interactions with coregulators or by influencing protein stability (Whitmarsh and Davis, 2007).

The upstream stimulatory factors (USF1 and USF2) are transcription factors that participate in the regulation of a large number of genes and especially USF2 appears to be crucial for the control of embryonic development, brain function, metabolism, iron homeostasis, fertility and growth whereas USF1 has roles in metabolism, as well as in the tanning and immune response (Sirito et al., 1998; Corre and Galibert, 2005). Furthermore, USFs seems to exhibit a tissue protective and tumor suppressive function in several cancer types (Ismail et al., 1999; Chen et al., 2006; Chang et al., 2005). Although the majority of mechanisms regulating USFs are largely unknown, phosphorylation appears to play an important role and the present review aims to summarize the current knowledge about the regulation of USFs by direct phosphorylation and the consequences for USF functions.

## THE UPSTREAM STIMULATORY FACTORS

Upstream stimulatory factors were identified in 1985 by their ability to regulate transcription of the adenovirus major late promoter (Carthew et al., 1985; Miyamoto et al., 1985; Sawadogo and Roeder, 1985). Sawadogo (1988) then succeeded in isolating two isoforms of the transcription factor, USF1 (43 kDa) and USF2 (44 kDa), from HeLa cells. Later, the human USF1 gene was located on chromosome 1q22–q23 (Shieh et al., 1993), whereas the human USF2 gene was found on chromosome 19q13 (Groenen et al., 1996). Both USF genes consist of 10 protein coding exons (Lin et al., 1994). By alternative splicing, two USF2 isoforms, USF2a (44 kDa) and USF2b (38 kDa), can be generated (Viollet et al., 1996) and have a gene regulatory function. The USF2 splice variant USF2b lacking the information encoded by exon 4 was shown to act as a dominant negative regulator of USF-dependent gene expression. Similar events were described for alternative splicing of exon 4 in USF1 mRNA (Gregor et al., 1990; Gao et al., 1997; Saito et al., 2003); again a novel USF1 variant affecting USF-dependent gene regulation was generated (Saito et al., 2003).

Although USF1 and USF2 are ubiquitously expressed, their ratio varies in different cell types (Sirito et al., 1994). Additionally, transcriptional regulation of USFs was discovered. For example, a *Helicobacter pylori* infection caused hypermethylation of the USF2 promoter in mice (Bussiere et al., 2001) and high glucose levels upregulated USF2 gene transcription via a cAMP-response element-binding protein (CREB) response element in the USF2 promoter (Shi et al., 2008).

*In vivo* the proteins appear mainly as USF1/USF2 heterodimers; homodimers are quite rare (Viollet et al., 1996). Experiments with USF knockout-mice revealed that there seems to be an asymmetrical cross-regulation between the two isotypes: USF1^–/–^ mice displayed enhanced USF2 expression, whereas USF2^–/–^ mice had less USF1 protein compared to wt mice (Sirito et al., 1998).

**FIGURE 1:**
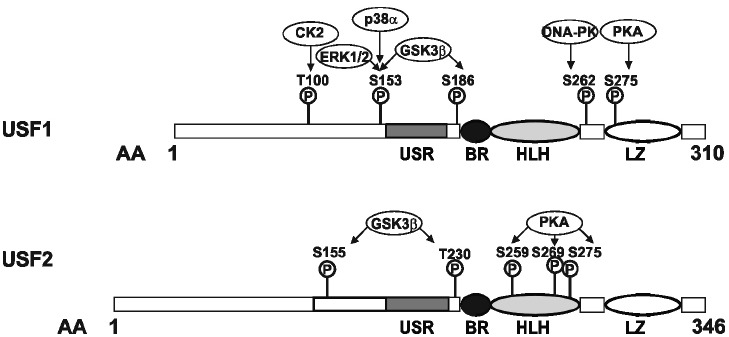
**Domain organization and phosphorylation sites within the transcription factors, upstream stimulating factor (USF).** USF1 and USF2 are b-HLH-LZ transcription factors with similar domain organization. The amino acid residues targeted by various kinases are indicated (see text for details). The conserved C-terminus of the USFs consists of a USF-specific region (USR, aa 158–183 in USF1; aa 194–219 in USF2), a basic region, a helix-loop-helix domain (BR-HLH, aa 199–254 in USF1, aa 235–290 in USF2), and a leucine zipper domain (LZ, 271–292 in USF1, 307–328 in USF2); the domain organization is given according to Sirito et al. (1992).

Upstream stimulatory factors are part of the basic helix-loop-helix leucine zipper (b-HLH-LZ) transcription factor family (Gregor et al., 1990) which also includes the oncoprotein c-Myc. The characteristic and highly conserved C-terminal b-HLH-LZ domain constitutes the conserved DBD, composed of a basic (b) region, followed by a HLH and a LZ motif. Both, USF1 and USF2, share about 70% identity within the b-HLH-LZ regions whereas the overall identity of the full length proteins is only about 44% (Sirito et al., 1994). Although the N-terminal regions share only a limited sequence homology, they contain a highly conserved USF-specific region (USR) which is located N-terminal from the basic region. The USR is supposed to play an important role in transcriptional activation (Groenen et al., 1996; Luo and Sawadogo, 1996b; Qyang et al., 1999). Furthermore, the USR as well as the basic region are important for mediating the nuclear localization of the transcription factor. Dimerization of the transcription factor is dependent on the HLH-LZ motif in the C-terminus of the protein (Luo and Sawadogo, 1996b; Figure 1).

Like the other members of the b-HLH transcription factor family, USFs bind in principle to E-boxes with a CANNTG core sequence in the promoters of their target genes (Baxevanis and Vinson, 1993). Whole genome ChIP-chip analyses in human HepG2 hepatoma cells showed that USF1/USF2 bind predominantly to CACGTGAC elements (Rada-Iglesias et al., 2008). In addition, USF2 but not USF1 was shown to bind also pyrimidine rich Inr elements in the core promoter of target genes (Roy et al., 1997). Due to the differences in the USF1 and USF2 N-termini it was suggested that the two isoforms have the ability to regulate different sets of genes and that they can be regulated in a different manner themselves (Luo and Sawadogo, 1996b).

## USF1 AND USF2 IN TISSUE PROTECTION AND CANCER

Given that USFs were identified as transcriptional regulators of the adenovirus major late promoter, it is not surprising that other viruses like HIV (Maekawa et al., 1991; Giacca et al., 1992; Sieweke et al., 1998), Varicella-zoster virus (Meier and Straus, 1995) and Epstein–Barr virus (Liu et al., 1996) use USFs during their replication cycle. Although these findings may suggest that USFs promote host invasion and tissue damage, it is also well accepted that USFs have rather a tissue protective role.

The role of USFs in tissue protection evolved first from their participation in the transcriptional regulation of the inflammatory genes and genes necessary for the immune response. Recent findings show that USF1 can inhibit inflammatory NF-*κ*B signaling by inducing expression of the gene tumor necrosis factor alpha (TNFα)-induced protein-3 (TNFAPI3, also known as TNFA1P2, OTUD7C, or A20; Tiruppathi et al., 2014). USF1 also promotes the immune response by stimulating expression of immunoglobulin light chain genes (Chang et al., 1992; Carter et al., 1997), the complement factor C4 gene (Galibert et al., 1997), and the major histocompatibility class I complex component β2-microglobulin (Gobin et al., 1999; Howcroft et al., 1999). Further, USF1 protects the skin from ultraviolet (UV) irradiation by inducing expression of pigmentation genes, and genes encoding factors necessary for nucleotide excision repair (NER; Baron et al., 2012). In addition to its proper function as a transcriptional regulator, recent findings unraveled a USF1/p53 crosstalk where in the presence of DNA damage, USF1 stabilizes p53 and induces cell cycle arrest (Bouafia et al., 2014). Moreover, USF2 was supposed to suppress cyclin-dependent kinase 4 (Cdk4) expression. Thus, the finding that USFs contribute to cell cycle regulation indicates their importance for cell protection, growth, and developmental processes.

Examination of USF-deficient mice illustrated that a minimum level of USF activity is required for embryonic development since USF1^–/–^/USF2^–/–^ mice die during embryogenesis. Both USFs seem to play a role in brain development because USF1^–/–^ as well as USF2^–/–^ mice were prone to spontaneous epileptic seizures. While USF1^–/–^ mice had no other obvious problems, more than half of the USF2^–/–^ pups died within two days after birth. USF2^–/–^ mice were about 20–40% smaller than their heterozygous siblings, male USF2^–/–^ had a drastically reduced lifespan and both male and female USF2^–/–^ mice had reduced fertility (Vallet et al., 1997, 1998; Sirito et al., 1998; Hadsell et al., 2003).

A recent study with USF2^–/–^ mice revealed that male USF2^–/–^ mice display dysregulated prostate growth and marked prostate hyperplasia already at a young age (Chen et al., 2006). This might be one of the reasons for the early death of male USF2^–/–^ mice. In line with this hypothesis, the authors have shown that the USF2 protein level of several prostate cancer cell lines is markedly decreased and that ectopic expression of USF2 in PC-3 prostate cancer cells inhibits their tumorigenicity. These data suggest that USF2 might have a tumor-suppressive function in prostate carcinogenesis (Chen et al., 2006).

That the action of USF2 as suppressor may be of special importance for prostate cancer was first highlighted by a study investigating hormone refractory prostate cancer samples. Half of the hormone refractory prostate cancer samples displayed a loss of chromosome 19q ter-q13.1 (Nupponen et al., 1998) which includes the region with the *Usf2* gene (Steingrimsson et al., 1995). A reintroduction of an intact human chromosome 19 into a tumorigenic prostate cell line reduced tumorigenicity in athymic nude mice (Astbury et al., 2001). In addition, USF2 has recently been found to be part of the prostatic factor complex as an androgen receptor cofactor (Kivinen et al., 2004) and it was found to be downregulated in human prostate cancer specimens (Chen et al., 2006). Recent investigations with a mouse *in vivo* xenograft model further substantiated the inhibitory role of USF2 since overexpression of USF2 in prostate cancer cells inhibited the tumorigenicity of these cells (Chen et al., 2006).

There are also other studies suggesting a role of USF2 in the development of cancer. In breast cancer cell lines, USF1 and USF2 were expressed, but a significant loss of transcriptional activity of both proteins was observed in approximately 50% of the transformed breast cell lines indicating that loss of USF function favors proliferation (Ismail et al., 1999). Furthermore, it was observed that USFs may have a potent growth-inhibitory effect and can antagonize the transforming function of the oncoproteins c-Myc and Ras in rat embryonic fibroblasts (Luo and Sawadogo, 1996a; Choe et al., 2005). Indeed, transfection of either USF1 or USF2 inhibited cellular transformation induced by c-Myc or activated Ras. In addition, USF2 also inhibited transformation in rat embryonic fibroblasts (REFs) induced by the adenovirus oncoprotein E1A, while USF1 did not, which highlights the broader inhibitory function of USF2 (Luo and Sawadogo, 1996a).

In line, many cancer cells including the prostate cancer cell line PC-3 (Ismail et al., 1999; Qyang et al., 1999) displayed a loss of USF2 transcriptional activity while it was active in non-tumorigenic cells.

Further, not only the oncogenes c-Myc and Ras, whose function could be counteracted by USF2 have been implicated in prostate cancer, but also tumor suppressor genes. Among the about 2500 genes which are supposed to be regulated by USFs (Rada-Iglesias et al., 2008) are those encoding the tumor suppressors adenomatous polyposis coli (APC), breast cancer 2 (BRAC2) or p53 (Reisman and Rotter, 1993; Davis et al., 1999; Jaiswal and Narayan, 2001; Wu et al., 2003). In addition, a number of genes important for cellular growth and metabolism like fatty acid synthetase (FAS), pyruvate carboxylase, pyruvate kinase, heme oxygenase (HO-1), plasminogen activator inhibitor-1 (PAI-1) or Cdk4 contain E-boxes that bind USF in their promoters (Reisman and Rotter, 1993; Jaiswal and Narayan, 2001; Wu et al., 2003; Wutthisathapornchai et al., 2014). Furthermore, the tumor-suppressor function of USFs was linked with their ability to repress the human telomerase reverse transcriptase (hTERT) expression in oral cancer cells; hTERT promotes immortalization and malignant transformation of cancer cells by stabilizing telomeric ends of chromosomes (Chang et al., 2005).

Contrary to the studies suggesting a tumor suppressor role for USF2 in carcinogenesis, there is one study indicating that USF2 has a pro-proliferative function in lung cancer cells (Ocejo-Garcia et al., 2005).

## INVOLVEMENT OF CO-FACTORS IN THE REGULATION OF USF ACTIVITY

There are some studies suggesting that the presence or availability of coactivators seems to play an important role in USFs activity. Indeed, both USF1 and USF2 were reported to associate with Fra1, a member of the b-Zip protein family, to promote transcription, demonstrating that cross-talk occurs between distant members of the protein family (Pognonec et al., 1997; Samoylenko et al., 2008). In addition, the bHLH transcription factor Cha could also constitute an important interactor, at least for USF1 where formation of the USF1/Cha heterodimer negatively affects USF-dependent transcription (Rodriguez et al., 2003). Although no proof has been obtained so far whether posttranslational modification events or just specific occurrence of these interactors regulate USF function, they may contribute to different cellular localization of USFs, in particular USF2. Since nuclear translocation of USF2 seems to be a tool for its regulation (Frenkel et al., 1998; Huang et al., 2008), the involvement of interacting factors in this process was proposed in experiments showing that USF2 was expressed and properly localized in the nucleus of Saos-2 cells, although USF2 was completely inactive in these cells (Qyang et al., 1999). Another study suggested the involvement of cofactors in the regulation of USF2 since it was polyubiquitinated and subsequently degraded by the proteasome in response to hypoxia (Jiang and Mendelson, 2005). In line with this, recent studies indicated that USF2 but not USF1 was required for hypoxia-dependent expression of genes regulated by the hypoxia-inducible transcription factor HIF2α. Thereby, USF2 binds to HIF2 target gene promoters, interacts with HIF2α, and recruits coactivators like CREB-binding protein (CBP) and p300 (Pawlus et al., 2012).

Moreover, USF2 in conjunction with the transcription factors YY1 and CCAAT/enhancer binding protein-β (C/EBPβ) affect expression of the cystic fibrosis transmembrane conductance regulator gene (CFTR) with YY1 being a repressor and USF2 being an activator. C/EBPβ appears to act as a switch; its phosphorylation favors the interaction between USF2 and YY1 thus blocking the inhibitory activity of the latter, in favor of C/EBPβ transactivation (Viart et al., 2013). Overall, this indicates that phosphorylation evokes an additional layer of complexity to the mechanisms influencing gene expression.

## PHOSPHORYLATION-DEPENDENT REGULATION OF USFs

So far, not much is known about the mechanisms contributing to the regulation of USFs in cancer and in general. Although phosphorylation appears to be a powerful and fast acting mechanism by which USFs function can be modulated, phosphorylation of USF proteins has not yet been investigated intensively and the number of studies showing direct phosphorylation of USF proteins by a kinase at certain residues is limited.

### INDIRECT EVIDENCE OF PHOSPHORYLATION-DEPENDENT REGULATION OF USFs

The first indirect evidence suggesting that USF1 might be a phosphoprotein came from the finding where phosphatase treatment of a purified USF1-like factor significantly reduced its DNA binding activity (Maekawa et al., 1991). Additionally, sphingosylphosphocholine (SPC) treatment of Swiss 3T3 fibroblasts resulted in enhanced USF1 DNA binding (Berger et al., 1998). Due to the fact that SPC can activate host kinases (Seufferlein and Rozengurt, 1995) it was suggested that SPC induced phosphorylation of USF1 (Berger et al., 1998) although this has not yet been shown. In addition, it has been reported that non-phosphorylated USF1 binds to the apolipoprotein A5 E-box and stimulates its transcription in HepG2 cells; insulin treatment reduced this, most likely via phosphorylation through the phosphatidylinositol-3-kinase (PI3K) pathway (Nowak et al., 2005). However, the localization and the relevance of the amino acid residues responsible for that effect were not shown, yet.

### DIRECT EVIDENCE OF PHOSPHORYLATION-DEPENDENT REGULATION OF USFs

More direct evidence for USF1 phosphorylation came from a study showing that USF1 is a phosphoprotein *in vivo*, and that the phosphorylated form of USF1 bound preferentially to an E-Box in the C4 complement gene promoter (Galibert et al., 1997).

#### Involvement of MAPK signaling in USF regulation

Mitogen-activated protein kinases (MAPKs) are a large family of serine/threonine kinases converting various extracellular signals, among them growth factors, mitogens or inflammatory cytokines, into intracellular responses through serial phosphorylation cascades, thus regulating cellular processes like proliferation, differentiation, development, stress responses, and apoptosis. Conventional MAPKs include extracellular signal-regulated kinase (ERK), ERK1 and ERK2 (p44/p42), c-Jun NH2-terminal kinase (JNK1/2/3), p38 MAPK (p38α/β/γ/δ) and ERK5. In addition, some atypical MAPKs such as ERK3/ERK4, NLK (nemo-like kinase), and ERK7/ERK8 have been identified but less is known about their regulation, substrate specificity and physiological functions (for review, see Coulombe and Meloche, 2007). From the kinases mentioned, only ERK1/2 and p38α have been described to phosphorylate USFs.

***ERK1/2 and USFs.*** Several studies suggested a role for ERK1/2 in the phosphorylation of USFs. In HepG2 cells ERK-mediated phosphorylation of USF1 could be detected following hepatocyte growth factor (HGF) treatment of the cells (Imagawa et al., 2006). Furthermore, ERK1/2-dependent activation of USFs was shown following *Helicobacter pylori* infection, though no direct phosphorylation was shown in these studies (Juttner et al., 2003). Further, it was found that USFs are downstream targets of the MAPK kinase (MEK)/ERK pathway in trigeminal ganglion neurons (Park et al., 2000). In all cases of the above mentioned studies, the authors claim that ERK1/2-dependent USF phosphorylation induces the transcriptional activity of USF (Park et al., 2000; Juttner et al., 2003; Imagawa et al., 2006).

More clear evidence for the importance of USF regulation by phosphorylation and their implication in cancer came from various studies investigating the expression of PAI-1. In many cancer types elevated PAI-1 levels can be detected and nowadays PAI-1 became a high evidence marker of a poor prognosis in breast cancer. Although the exact mechanisms by which PAI-1 influences tumor growth and dissemination remain partially controversial, it is evident that PAI-1 exerts its effects not only on fibrinolysis but also on cell attachment, migration and angiogenesis (Andreasen et al., 1997; Dimova et al., 2004; Dellas and Loskutoff, 2005; Dimova and Kietzmann, 2008).

Plasminogen activator inhibitor-1 gene expression is tightly regulated and can be induced by a variety of hormones (insulin, glucagon, glucocorticoids, angiotensin II), growth factors (insulin-like growth factor, IGF-I; transforming growth factor-β, TGF-β), cytokines (interleukin-1, IL-1), TNFα, metabolic signals (glucose) and environmental signals (hypoxia; Dimova and Kietzmann, 2008).

In this context USFs seem to be quite important in mediating the response of several signals to the PAI-1 promoter. USF1 was shown to stimulate PAI-1 expression when being induced by serum (White et al., 2000) and by wounding (Providence et al., 2002). Thereby, phosphorylation of USF1 seemed to be required for DNA binding (Cheung et al., 1999; Providence et al., 2002). USF2 repressed PAI-1 expression in primary rat hepatocytes (Samoylenko et al., 2001); likewise USF2 mediated suppression of PAI-1 expression in response to the polyphenol quercetin in human endothelial cells (Olave et al., 2010); however, it induced PAI-1 expression in human and in rat hepatoma cell lines, indicating that the effect of USF2 on PAI-1 expression is cell-type specific (Dimova and Kietzmann, 2006). In this context it was shown that binding of USFs is important for HGF-mediated induction of PAI-1 gene expression (Imagawa et al., 2006). Thereby, USF1 was phosphorylated through the MAPK pathway which appears to be also important in mediating PAI-1 transcription in response to TGF-β (Riccio et al., 1992; Allen et al., 2005). In human epidermal keratinocytes USF1 and USF2 occupy the human PAI promoter in a dynamical manner as a function of growth state. While USF1 binds in quiescent cells and represses PAI-1 expression, USF2 stimulates expression after serum-stimulated binding to the human PAI promoter (Qi et al., 2006). In this context MAPK-mediated phosphorylation of USF1 at Thr153 is required for suppression of PAI-1 expression (Qi et al., 2006). Recent studies suggested that TGF-β-induced PAI-1 expression is dependent on epidermal growth factor receptor (EGFR)-mediated stimulation of the MEK/ERK pathway and that USF1 is phosphorylated by this signaling cascade (Kutz et al., 2006; Samarakoon et al., 2009).

Overall, these studies indicate that USF1 can be phosphorylated via the ERK1/2 pathway while less is known about the role of ERK1/2 on USF2. In addition, cell-type specific components need to be considered and to be discovered to fully understand the role of phosphoUSF1 in PAI-1 expression and cancer.

***P38 and USFs.*** The p38 MAPKs were identified as modulators of TNF signaling (Sabio and Davis, 2014) but meanwhile p38 MAPKs were found to be activated by many other stimuli including oxidative and chemical stress, osmotic and heat shock as well as hormones. There are at least four p38 kinases named α, β, γ, and δ, from which the p38α enzyme is the best characterized (Sabio and Davis, 2014). A study describing that USF1 is involved in the UV stress response in COS7 and B16 melanoma cells was the first showing that not JNK but the stress-responsive p38α acts as an USF1-phosphorylating kinase. The p38α-mediated phosphorylation occurred in the N-terminal part of USF1 at Thr153 and induced transcription of the tyrosinase gene (Galibert et al., 2001). The tyrosinase is the rate-controlling enzyme for the production of melanin and thus is necessary for pigmentation and the tanning response. Moreover, USF1 seems to be also critical for the transcriptional induction of other genes like pro-opiomelanocortin, and melanocortin 1 receptor which failed to be activated following UV stimulation in USF1^–/–^ melanocytes (Corre et al., 2004).

Since UV light is not only known to induce a tanning but also a DNA damage response (Wu et al., 2003; Ditch and Paull, 2012), it is possible that UV irradiation promotes the interaction of USF transcription factors with genes involved in NER of DNA. Indeed, recent findings indicate that the proximal promoters of HR23A (homologues of the yeast RAD23) and CSA (excision repair cross-complementation group 8) contained E-boxes and were regulated by USFs (Baron et al., 2012). Overall, these findings support the view that phosphorylation of USF-1 by p38 can contribute to the protection of skin against UV light-induced DNA damage.

In this regard the phosphorylation of USF1 by p38α may be important in tissue protection against skin cancer. Together, these findings again underline the potential tumor suppressive aspects of USFs.

#### Involvement of other kinases

***Cdk2 and USFs.*** The cell cycle is regulated by the interplay of a catalytic Cdk and its regulatory cyclin. The Cdk2 is an important regulator within this process. It is critical for G1-S transition within the cell cycle, the initiation of DNA synthesis, but also for modulating G2 progression. It appears to be crucial for cancerogenesis since it is orchestrating a fine balance between cellular proliferation, cell death, and DNA repair (Satyanarayana and Kaldis, 2009). In this respect one study indicated that USF1 can be phosphorylated by Cdk2 within a region encompassing the amino acids from 143 to 197; this phosphorylation event increased the DNA-binding activity of the transcription factor (Cheung et al., 1999). These data may be of importance in the context of cancer cell proliferation where this type of regulation may counterbalance the effects of c-Myc. Interestingly, c-Myc can also be phosphorylated by Cdk2 (Hydbring and Larsson, 2010a,b); in contrast to USF1, Cdk2 phosphorylated c-Myc displays reduced DNA-binding activity. In this respect phospho-USF1 may outcompete c-Myc and coordinate a cellular response where USF1 would be anti-proliferative.

***Protein kinase C (PKC) and USFs.*** PKC is a superfamily of serine/threonine kinases consisting of about 10 to 12 isoforms divided in three subfamilies based on their second messenger requirements (for review, see Altman and Kong, 2014; Gomez-Cambronero, 2014). With respect to USFs, it was reported that PKC can phosphorylate rat USF1 *in vitro* and *in vivo* in neonatal rat ventricular myocytes (Xiao et al., 2002). In that setting PKC-mediated phosphorylation of USF1 increased the binding activity to the cardiac α-myosin heavy chain promoter (Xiao et al., 2002). In the study of Xiao et al. (2002) a rat brain extract containing a mixture of PKC isoforms α, β, and γ was used. Since distinct PKC isoforms differ in their substrate specificity (Casabona, 1997) it is possible that the various PKC isoforms may confer different effects. Although no data for USF2 are available, these data imply that USFs respond to PKC signaling. However, it remains open to what extend the different isoforms and cell-type specific factors contribute to their function. Hence, further studies would be necessary to better understand the role of PKCs and USF phosphorylation in the context of cancer.

***DNA-dependent protein kinase (DNA-PK) and USFs.*** The DNA-PK is a heterotrimer consisting of the DNA-dependent protein kinase catalytic subunit (PRKDC) and a dimer composed of the Ku p70 and Ku p86 (XRCC6-XRCC5) proteins. It acts as a serine/threonine-protein kinase that usually responds as a molecular sensor for DNA damage. It is involved in the non-homologous end joining (NHEJ) double-strand break (DSB) DNA repair. There, it usually associates with the DNA-bound Ku heterodimer, but it can also bind to and be activated by free DNA. Apart from phosphorylating histone H2A it was found to modulate transcription factors like c-Jun, p53, or c-Myc and contributes to the determination of the circadian period length by affecting Cry1 phosphorylation (Deriano and Roth, 2013; Davis et al., 2014).

Moreover, a recent study implicated a function of DNA-PK in insulin signaling. This study proposed a model where feeding or insulin activates DNA-PK by dephosphorylation caused by the action of protein phosphatase 1 (PP1). As a consequence DNA-PK phosphorylates USF1 at Ser262 in the b-HLH-LZ domain. Phosphorylation of USF1 allows p300 associated factor (P/CAF) recruitment and subsequent acetylation of USF1 at Lys237. This acetylation event then enhanced the transcriptional activity of USF1 with respect to the FAS promoter (Wong and Sul, 2009).

Recently it has become obvious that beside its metabolic functions in lipogenesis, FAS also seems to play an important role in carcinogenesis. It was observed that FAS expression was elevated in breast, prostate, colon, ovary, endometrium, and thyroid tumor tissues compared to normal tissue. FAS expression thereby seems to confer a growth and/or survival advantage and is associated with a poor prognosis (Kuhajda, 2000, 2006). The findings that insulin via DNA-PK can contribute to enhanced FAS expression would argue against a tumor suppressive role of USF1; however, in contrast to normal tissue where FAS is mainly regulated by diet, in cancer cells FAS seems to be regulated also through the MAPK and the PI3K pathways with additional intracellular players (Van de Sande et al., 2002; Yang et al., 2002). Thus, other factors than USFs may have a more dominant role for the regulation of FAS expression in cancer cells.

***Protein kinase A (PKA) and USFs.*** PKA, a cAMP-dependent protein kinase, is an ubiquitous serine/threonine kinase involved in a wide range of cellular processes such as transcription, metabolism, cell cycle, and apoptosis (Arencibia et al., 2013; Stratakis, 2013). The intracellular cAMP level regulates cellular responses by altering the activity of PKA. PKA was found to be involved in USF regulation in the bovine system. In a study investigating the effect of forskolin and PKA on the bovine prostaglandin G/H-2 synthase promoter, it was found that overexpression of PKA enhanced USF DNA binding in bovine granulosa cells. While bovine USF1 contains two putative PKA sites, bovine USF2 contains three PKA phosphorylation sites. Interestingly, only mutation of S275 in bovine USF1 reduced but did not abolish its transactivation capacity whereas mutation of the second putative PKA site S262 had no effect in the context of the prostaglandin G/H-2 promoter (Sayasith et al., 2005). By contrast, when single mutations were introduced at the putative PKA phosphorylation sites S259, S269, and S275 in USF2, this reduced but did not abolish its transactivation capacity (Sayasith et al., 2005).

The cAMP-mediated pathway and a correct functioning PKA cascade was found to be involved in the regulation of the cell cycle via cyclin D. A defect in this cascade would again link cAMP, PKA, and USFs with the development of cancer. However, the link appears to be controversial. Although a reduction in cAMP has an anti-proliferative effect on colorectal cancer cells (Löffler et al., 2008), an increase in the PKA type I isozyme induces a non-tumorigenic phenotype in lung cancer cells (Porter et al., 2001). To what extend USFs are involved in this response has not yet been unraveled and further studies would be needed to gain more insight into the connection of PKA and USF regulation during cancerogenesis.

***Glycogen synthase kinase-3 (GSK-3) and USFs.*** GSK3 is a serine/threonine kinase that was first identified as a negative regulator of glycogen synthesis; inhibition is achieved through phosphorylation of glycogen synthase (Embi et al., 1980; Woodgett, 1990). Since its initial discovery, GSK3 was found to be a key player in several signal transduction pathways, such as the PI3K/Akt pathway, the Wnt/β-catenin pathway or the Hedgehog signaling pathway. Due to these multiple involvements, dysregulation of GSK3 has been implicated in the pathogenesis of human diseases, including type-2 diabetes, bipolar disorders, inflammation, Alzheimer’s disease, and cancer (reviewed by Frame and Cohen, 2001; Grimes and Jope, 2001). Two isoforms, GSK3α (51 kDa) and GSK3β (47 kDa), have been identified in mammals. Despite their homology in the catalytic domain (98%), they significantly differ in their N- and C-terminal parts and do not have entirely overlapping roles in metabolism (reviewed in Doble and Woodgett, 2003; Force and Woodgett, 2009).

While GSK3β promoted cancer development and growth in some types of cancer, it had a tumor suppressor function in other types since a decrease in GSK3β function or expression was observed in these tumors (Luo, 2009). In line with the latter findings and in the context of phosphorylation-dependent regulation of USFs, a recently published article reports GSK3β-induced phosphorylation of USF1 following inhibition of the PI3K pathway (Terragni et al., 2011). By means of mass spectrometry analysis of GSK3β-phosphorylated human recombinant USF1 protein, the authors identified Thr153 and Ser186 as the phosphorylated amino acids. While phosphorylation of Thr153 was important for the transcriptional activation of genes promoting apoptosis and cell cycle arrest, phosphorylation of Ser186 had no effect (Terragni et al., 2011).

In addition, GSK3β acts also as an USF2-phosphorylating kinase. The phosphorylation sites within USF2 could be mapped to serine 155 and threonine 230. *In silico* analyses of the 3-dimensional structure revealed that phosphorylation of USF2 by GSK3β converts it to a more open conformation which may influence transactivity, DNA binding and target gene expression. Indeed, experiments with GSK3β-deficient cells revealed that USF2 transactivity, DNA binding and target gene expression were reduced upon lack of GSK3β. Further, experiments with USF2 variants mimicking GSK3β phosphorylated USF2 in GSK3β-deficient cells showed that phosphorylation of USF2 by GSK3β did not affect cell proliferation but increased cell migration (Horbach et al., 2014). Together, these studies indicate that GSK3β is an important modulator of USF function; however, the exact function of both, GSK3β and USF2 in cancerogenesis appears to be variable and may depend on the cellular context. Some studies support the idea of GSK3β and USF2 being tumor suppressive (Luo and Sawadogo, 1996a; Aberle et al., 1997), other studies show a cancer promoting effect (Ocejo-Garcia et al., 2005; Landa et al., 2009). Although no study has yet correlated the activity of GSK3β with the activity of USF2 in a certain tumor setting, the findings of the latter study would favor the tumor promoting aspects of GSK3β and USF2 since GSK3β activated USF2 enhanced cell migration which may be important in terms of tumor cell metastasis. However, different growth conditions, the cellular or tissue-specific context may influence the activity of USF2.

In summary, more studies on the regulation of the transcription factor USF2 are necessary to understand the mechanisms by which it affects the development of different types of cancer.

***CK2 and USFs.*** Protein kinase CK2 (formerly known as casein kinase II) is considered to act as messenger-independent protein serine/threonine kinase. CK2 is a tetrameric enzyme consisting of two catalytic α and α′ subunits and two non-catalytic β-subunits. Interestingly, the majority of CK2 targets are proteins involved in signaling, protein synthesis and transcriptional regulation (Montenarh, 2010). Over the last 10 years a number of transcription factors have been detected which are phosphorylated by CK2; phosphorylation enhances transcriptional activity of activating transcription factor 4 (ATF4; Ampofo et al., 2013), HIF1α (Mottet et al., 2005), upstream binding factor (UBF; Lin et al., 2006) and FoxM1c (Wierstra, 2011) and reduces transcriptional activity of PDX-1 (Welker et al., 2013) and Chop (Schneider et al., 2012) just to mention a few. A recent report combining *in silico*, *in vitro* and *in vivo* assays added USF1 but not USF2 to that list. Further, by using USF1 deletion mutants and point mutants that study identified threonine 100 as the major phosphorylation site for CK2. In addition, inhibition of CK2 with a specific inhibitor enhanced binding of USF1 to USF2, i.e., heterodimerization. Furthermore, transactivation studies showed that inhibition of CK2-dependent phosphorylation of USF1 stimulated transcription from the pancreatic glucokinase promoter as well as the FAS promoter but not from the HO-1 promoter (Lupp et al., 2014). Thus, this study shows that phosphorylation of USF1 by CK2 modulates two functionally important properties of USF1, namely heterodimerization and transactivation.

## CONCLUSION

Transcription factors are critical components within signal transduction pathways and via regulating the expression of various genes they are involved in various aspects of cellular functions including regulation of cell growth and cell death. Therefore, disturbances within the proper function of transcription factors may be related to tumorigenesis and cancer. Phosphorylation events are fundamental processes achieved through the activity of various protein kinases which allow immediate control of protein activity including transcription factor function. Thus, the knowledge of the signaling pathways and the involved kinases are of immediate importance to understand the regulation and proper function of transcription factors. The transcription factors USF1 and USF2 have been shown to be subject of phosphorylation by different kinases; though most of the studies refer to USF1. In addition to their involvement in embryonic development, brain function, metabolism, iron homeostasis, fertility, pigmentation, and the immune response it became also evident that USFs affect tumorigenesis. Since kinase inhibitors became a feasible therapeutic approach in anti-tumor therapy within the last decade, they may be considered also to be of use in terms of the role of USFs in tumorigenesis and cancer. However, a concise view about the impact of USF’s phosphorylation during tumorigenesis has not been reached. The different phosphorylation events need to be considered in a more specific cellular context and in conjunction with USF interacting proteins. This may be of special importance since the phosphorylation events together with cell-specific USF interacting partner molecules may then render USFs either into a tumor suppressor or tumor promoter. Thus, more studies in particular considering interacting partners and cell-type specific effectors are necessary to further define the mode by which USFs affect tissue injury, inflammation, and tumorigenesis.

### Conflict of Interest Statement

The authors declare that the research was conducted in the absence of any commercial or financial relationships that could be construed as a potential conflict of interest.

## References

[B1] Aberle H. Bauer A. Stappert J. Kispert A. Kemler R. (1997). β-catenin is a target for the ubiquitin-proteasome pathway. EMBO J. 16, 3797–3804. 10.1093/emboj/16.13.3797 9233789 PMC1170003

[B2] Adhikary S. Eilers M. (2005). Transcriptional regulation and transformation by Myc proteins. Nat. Rev. Mol. Cell Biol. 6, 635–645. 10.1038/nrm1703 16064138

[B3] Allen R. R. Qi L. Higgins P. J. (2005). Upstream stimulatory factor regulates E box-dependent PAI-1 transcription in human epidermal keratinocytes. J. Cell. Physiol. 203, 156–165. 10.1002/jcp.20211 15372465

[B4] Altman A. Kong K. F. (2014). Protein kinase C inhibitors for immune disorders. Drug Discov. Today 19, 1217–1221. 10.1016/j.drudis.2014.05.008 24892801 PMC4138286

[B5] Ampofo E. Sokolowsky T. Götz C. Montenarh M. (2013). Functional interaction of protein kinase CK2 and activating transcription factor 4 (ATF4), a key player in the cellular stress response. Biochim. Biophys. Acta 1833, 439–451. 10.1016/j.bbamcr.2012.10.025 23123191

[B6] Andreasen P. A. Kjoller L. Christensen L. Duffy M. J. (1997). The urokinase-type plasminogen activator system in cancer metastasis: a review. Int. J. Cancer 72, 1–22 10.1002/(SICI)1097-0215(19970703)72:1<1::AID-IJC1>3.0.CO;2-Z 9212216

[B7] Arencibia J. M. Pastor-Flores D. Bauer A. F. Schulze J. O. Biondi R. M. (2013). AGC protein kinases: from structural mechanism of regulation to allosteric drug development for the treatment of human diseases. Biochim. Biophys. Acta 1834, 1302–1321. 10.1016/j.bbapap.2013.03.010 23524293

[B8] Astbury C. Jackson-Cook C. K. Culp S. H. Paisley T. E. Ware J. L. (2001). Suppression of tumorigenicity in the human prostate cancer cell line M12 via microcell-mediated restoration of chromosome 19. Genes Chromosomes Cancer 31, 143–155. 10.1002/gcc.1128 11319802

[B9] Bannister A. J. Miska E. A. (2000). Regulation of gene expression by transcription factor acetylation. Cell. Mol. Life Sci. 57, 1184–1192. 10.1007/PL00000758 11028911 PMC11147133

[B10] Baron Y. Corre S. Mouchet N. Vaulont S. Prince S. Galibert M. D. (2012). USF-1 is critical for maintaining genome integrity in response to UV-induced DNA photolesions. PLoS Genet. 8:e1002470. 10.1371/journal.pgen.1002470 22291606 PMC3266871

[B11] Baxevanis A. D. Vinson C. R. (1993). Interactions of coiled coils in transcription factors: where is the specificity? Curr. Opin. Genet. Dev. 3, 278–285 10.1016/0959-437X(93)90035-N 8504253

[B12] Berger A. Cultaro C. M. Segal S. Spiegel S. (1998). The potent lipid mitogen sphingosylphosphocholine activates the DNA binding activity of upstream stimulating factor (USF), a basic helix-loop-helix-zipper protein. Biochim. Biophys. Acta 1390, 225–236 10.1016/S0005-2760(97)00180-X 9507145

[B13] Bouafia A. Corre S. Gilot D. Mouchet N. Prince S. Galibert M. D. (2014). p53 requires the stress sensor USF1 to direct appropriate cell fate decision. PLoS Genet. 10:e1004309. 10.1371/journal.pgen.1004309 24831529 PMC4022457

[B14] Bussiere M. Vance J. E. Campenot R. B. Vance D. E. (2001). Compartmentalization of choline and acetylcholine metabolism in cultured sympathetic neurons. J. Biochem. (Tokyo) 130, 561–568 10.1093/oxfordjournals.jbchem.a003019 11574076

[B15] Carter R. S. Ordentlich P. Kadesch T. (1997). Selective utilization of basic helix-loop-helix-leucine zipper proteins at the immunoglobulin heavy-chain enhancer. Mol. Cell. Biol. 17, 18–23. 8972181 10.1128/mcb.17.1.18 PMC231725

[B16] Carthew R. W. Chodosh L. A. Sharp P. A. (1985). An RNA polymerase II transcription factor binds to an upstream element in the adenovirus major late promoter. Cell 43, 439–448 10.1016/0092-8674(85)90174-6 4075400

[B17] Casabona G. (1997). Intracellular signal modulation: a pivotal role for protein kinase C. Prog. Neuropsychopharmacol. Biol. Psychiatry 21, 407–425 10.1016/S0278-5846(97)00011-0 9153066

[B18] Chang J. T. C. Yang H. T. Wang T. C. V. Cheng A. J. (2005). Upstream stimulatory factor (USF) as a transcriptional suppressor of human telomerase reverse transcriptase (hTERT) in oral cancer cells. Mol. Carcinog. 44, 183–192. 10.1002/mc.20129 16010690

[B19] Chang L. A. Smith T. Pognonec P. Roeder R. G. Murialdo H. (1992). Identification of USF as the ubiquitous murine factor that binds to and stimulates transcription from the immunoglobulin lambda 2-chain promoter. Nucleic Acids Res. 20, 287–293. 10.1093/nar/20.2.287 1741254 PMC310368

[B20] Chen N. Szentirmay M. N. Pawar S. A. Sirito M. Wang J. Wang Z. (2006). Tumor-suppression function of transcription factor USF2 in prostate carcinogenesis. Oncogene 25, 579–587. 10.1038/sj.onc.1209079 16186802

[B21] Cheung E. Mayr P. Coda-Zabetta F. Woodman P. G. Boam D. (1999). DNA-binding activity of the transcription factor upstream stimulatory factor 1 (USF-1) is regulated by cyclin-dependent phosphorylation. Biochem. J. 344, 145–152. 10.1042/0264-6021:3440145 10548544 PMC1220624

[B22] Choe C. Chen N. Sawadogo M. (2005). Decreased tumorigenicity of c-Myc-transformed fibroblasts expressing active USF2. Exp. Cell Res. 302, 1–10. 10.1016/j.yexcr.2004.08.013 15541720

[B23] Corre S. Galibert M. D. (2005). Upstream stimulating factors: highly versatile stress? Responsive transcription factors. Pigment Cell Res. 18, 337–348. 10.1111/j.1600-0749.2005.00262.x 16162174

[B24] Corre S. Primot A. Sviderskaya E. Bennett D. C. Vaulont S. Goding C. R. (2004). UV-induced expression of key component of the tanning process, the POMC and MC1R genes, is dependent on the p-38-activated upstream stimulating factor-1 (USF-1). J. Biol. Chem. 279, 51226–51233. 10.1074/jbc.M409768200 15358786

[B25] Coulombe P. Meloche S. (2007). Atypical mitogen-activated protein kinases: structure, regulation and functions. Biochim. Biophys. Acta 1773, 1376–1387. 10.1016/j.bbamcr.2006.11.001 17161475

[B26] Davis A. J. Chen B. P. Chen D. J. (2014). DNA-PK: a dynamic enzyme in a versatile DSB repair pathway. DNA Repair (Amst.) 17, 21–29. 10.1016/j.dnarep.2014.02.020 24680878 PMC4032623

[B27] Davis P. L. Miron A. Andersen L. M. Iglehart J. D. Marks J. R. (1999). Isolation and initial characterization of the BRCA2 promoter. Oncogene 18, 6000–6012. 10.1038/sj.onc.1202990 10557089

[B28] Dellas C. Loskutoff D. J. (2005). Historical analysis of PAI-1 from its discovery to its potential role in cell motility and disease. Thromb. Haemost. 93, 631–640. 10.1160/TH05-01-0033 15841306

[B29] Deriano L. Roth D. B. (2013). Modernizing the nonhomologous end-joining repertoire: alternative and classical NHEJ share the stage. Annu. Rev. Genet. 47, 433–455. 10.1146/annurev-genet-110711-155540 24050180

[B30] Desterro J. M. P. Rodriguez M. S. Hay R. T. (2000). Regulation of transcription factors by protein degradation. Cell. Mol. Life Sci. 57, 1207–1219. 10.1007/PL00000760 11028913 PMC11146932

[B31] Dimova E. Y. Kietzmann T. (2006). Cell type-dependent regulation of the hypoxia-responsive plasminogen activator inhibitor-1 gene by upstream stimulatory factor-2. J. Biol. Chem. 281, 2999–3005. 10.1074/jbc.M512078200 16330554

[B32] Dimova E. Y. Kietzmann T. (2008). Metabolic, hormonal and environmental regulation of plasminogen activator inhibitor-1 (PAI-1) expression: lessons from the liver. Thromb. Haemost. 100, 992–1006. 10.1160/TH08-07-0490 19132222

[B33] Dimova E. Y. Samoylenko A. Kietzmann T. (2004). Oxidative stress and hypoxia: implications for plasminogen activator inhibitor-1 expression. Antioxid. Redox Signal. 6, 777–791. 10.1089/1523086041361596 15242559

[B34] Ditch S. Paull T. T. (2012). The ATM protein kinase and cellular redox signaling: beyond the DNA damage response. Trends Biochem. Sci. 37, 15–22. 10.1016/j.tibs.2011.10.002 22079189 PMC3259275

[B35] Doble B. W. Woodgett J. R. (2003). GSK-3: tricks of the trade for a multi-tasking kinase. J. Cell Sci. 116, 1175–1186. 10.1242/jcs.00384 12615961 PMC3006448

[B36] Embi N. Rylatt D. B. Cohen P. (1980). Glycogen synthase kinase-3 from rabbit skeletal muscle. Separation from cyclic-AMP-dependent protein kinase and phosphorylase kinase. Eur. J. Biochem. FEBS 107, 519–527 10.1111/j.1432-1033.1980.tb06059.x 6249596

[B37] Force T. Woodgett J. R. (2009). Unique and overlapping functions of GSK-3 isoforms in cell differentiation and proliferation and cardiovascular development. J. Biol. Chem. 284, 9643–9647. 10.1074/jbc.R800077200 19064989 PMC2665084

[B38] Frame S. Cohen P. (2001). GSK3 takes centre stage more than 20 years after its discovery. Biochem. J. 359, 1–16. 10.1042/0264-6021:3590001 11563964 PMC1222116

[B39] Frenkel S. Kay G. Nechushtan H. Razin E. (1998). Nuclear translocation of upstream stimulating factor 2 (USF2) in activated mast cells: a possible role in their survival. J. Immunol. 161, 2881–2887. 9743349

[B40] Galibert M. D. Boucontet L. Goding C. R. Meo T. (1997). Recognition of the E-C4 element from the C4 complement gene promoter by the upstream stimulatory factor-1 transcription factor. J. Immunol. 159, 6176–6183. 9550420

[B41] Galibert M. D. Carreira S. Goding C. R. (2001). The Usf-1 transcription factor is a novel target for the stress-responsive p38 kinase and mediates UV-induced Tyrosinase expression. EMBO J. 20, 5022–5031. 10.1093/emboj/20.17.5022 11532965 PMC125271

[B42] Gao E. Wang Y. Alcorn J. L. Mendelson C. R. (1997). The basic helix-loop-helix-zipper transcription factor USF1 regulates expression of the surfactant protein-A gene. J. Biol. Chem. 272, 23398–23406. 10.1074/jbc.272.37.23398 9287355

[B43] Giacca M. Gutierrez M. I. Menzo S. di Fagagna F. D. Falaschi A. (1992). A human binding site for transcription factor USF/MLTF mimics the negative regulatory element of human immunodeficiency virus type 1. Virology 186, 133–147 10.1016/0042-6822(92)90067-Y 1727595

[B44] Gill G. (2005). Something about SUMO inhibits transcription. Curr. Opin. Genet. Dev. 15, 536–541. 10.1016/j.gde.2005.07.004 16095902

[B45] Gobin S. J. van Zutphen M. Woltman A. M. van den Elsen P. J. (1999). Transactivation of classical and nonclassical HLA class I genes through the IFN-stimulated response element. J. Immunol. 163, 1428–1434. 10415043

[B46] Gomez-Cambronero J. (2014). Phospholipase D in cell signaling: from a myriad of cell functions to cancer growth and metastasis. J. Biol. Chem. 289, 22557–22566. 10.1074/jbc.R114.574152 24990944 PMC4132763

[B47] Gregor P. D. Sawadogo M. Roeder R. G. (1990). The adenovirus major late transcription factor USF is a member of the helix-loop-helix group of regulatory proteins and binds to DNA as a dimer. Genes Dev. 4, 1730–1740. 10.1101/gad.4.10.1730 2249772

[B48] Grimes C. A. Jope R. S. (2001). The multifaceted roles of glycogen synthase kinase 3β in cellular signaling. Prog. Neurobiol. 65, 391–426 10.1016/S0301-0082(01)00011-9 11527574

[B49] Groenen P. Garcia E. Debeer P. Devriendt K. Fryns J. P. Ven W. J. M. (1996). Structure, sequence, and chromosome 19 localization of human USF2 and its rearrangement in a patient with multicystic renal dysplasia. Genomics 38, 141–148. 10.1006/geno.1996.0609 8954795

[B50] Hadsell D. L. Bonnette S. George J. Torres D. Klementidis Y. Gao S. (2003). Diminished milk synthesis in upstream stimulatory factor 2 null mice is associated with decreased circulating oxytocin and decreased mammary gland expression of eukaryotic initiation factors 4E and 4G. Mol. Endocrinol. 17, 2251–2267. 10.1210/me.2002-0031 12907752

[B51] Horbach T. Chi F. Goetz C. Sharma S. Juffer A. H. Dimova E. Y. (2014). GSK3β-dependent phosphorylation alters DNA binding, transactivity and half-life of the transcription factor USF2. PLoS ONE 9:e107914. 10.1371/journal.pone.0107914 25238393 PMC4169611

[B52] Howcroft T. K. Murphy C. Weissman J. D. Huber S. J. Sawadogo M. Singer D. S. (1999). Upstream stimulatory factor regulates major histocompatibility complex class I gene expression: the U2ΔE4 splice variant abrogates E-box activity. Mol. Cell. Biol. 19, 4788–4797. 10373528 10.1128/mcb.19.7.4788 PMC84277

[B53] Huang Y. H. Huang C. C. Chuang J. H. Hsieh C. S. Lee S. Y. Chen C. L. (2008). Upstream stimulatory factor 2 is implicated in the progression of biliary atresia by regulation of hepcidin expression. J. Pediatr. Surg. 43, 2016–2023. 10.1016/j.jpedsurg.2008.03.037 18970934

[B54] Hydbring P. Larsson L. G. (2010a). Tipping the balance: Cdk2 enables Myc to suppress senescence. Cancer Res. 70, 6687–6691. 10.1158/0008-5472.CAN-10-1383 20713526

[B55] Hydbring P. Larsson L. G. (2010b). Cdk2: a key regulator of the senescence control function of Myc. Aging 2, 244–250. 20445224 10.18632/aging.100140 PMC2881513

[B56] Imagawa S. Fujii S. Dong J. Furumoto T. Kaneko T. Zaman T. (2006). Hepatocyte growth factor regulates E box-dependent plasminogen activator inhibitor type 1 gene expression in HepG2 liver cells. Arterioscler. Thromb. Vasc. Biol. 26, 2407–2413. 10.1161/01.ATV.0000240318.61359.e3 16902162

[B57] Ismail P. M. Lu T. Sawadogo M. (1999). Loss of USF transcriptional activity in breast cancer cell lines. Oncogene 18, 5582–5591. 10.1038/sj.onc.1202932 10523835

[B58] Jaiswal A. S. Narayan S. (2001). Upstream stimulating factor-1 (USF1) and USF2 bind to and activate the promoter of the *adenomatous polyposis coli* (APC) tumor suppressor gene. J. Cell. Biochem. 81, 262–277 10.1002/1097-4644(20010501)81:2<262::AID-JCB1041>3.0.CO;2-R 11241666

[B59] Jiang B. Mendelson C. R. (2005). O_2_ enhancement of human trophoblast differentiation and *hCYP19* (aromatase) gene expression are mediated by proteasomal degradation of USF1 and USF2. Mol. Cell. Biol. 25, 8824–8833. 10.1128/MCB.25.20.8824-8833.2005 16199862 PMC1265767

[B60] Juttner S. Cramer T. Wessler S. Walduck A. Gao F. Schmitz F. (2003). *Helicobacter pylori* stimulates host cyclooxygenase-2 gene transcription: critical importance of MEK/ERK-dependent activation of USF1/-2 and CREB transcription factors. Cell. Microbiol. 5, 821–834. 10.1046/j.1462-5822.2003.00324.x 14531897

[B61] Kivinen A. Patrikainen L. Kurkela R. Porvari K. Vihko P. (2004). USF2 is connected to GAAAATATGATA element and associates with androgen receptor-dependent transcriptional regulation in prostate. Prostate 59, 190–202. 10.1002/pros.20015 15042619

[B62] Kuhajda F. P. (2000). Fatty-acid synthase and human cancer: new perspectives on its role in tumor biology. Nutrition 16, 202–208 10.1016/S0899-9007(99)00266-X 10705076

[B63] Kuhajda F. P. (2006). Fatty acid synthase and cancer: new application of an old pathway. Cancer Res. 66, 5977–5980. 10.1158/0008-5472.CAN-05-4673 16778164

[B64] Kutz S. M. Higgins C. E. Samarakoon R. Higgins S. P. Allen R. R. Qi L. (2006). TGF-β 1-induced PAI-1 expression is E box/USF-dependent and requires EGFR signaling. Exp. Cell Res. 312, 1093–1105. 10.1016/j.yexcr.2005.12.027 16457817

[B65] Landa I. Ruiz-Llorente S. Montero-Conde C. Inglada-Perez L. Schiavi F. Leskela S. (2009). The variant rs1867277 in *FOXE1* gene confers thyroid cancer susceptibility through the recruitment of USF1/USF2 transcription factors. PLoS Genet. 5:e1000637. 10.1371/journal.pgen.1000637 19730683 PMC2727793

[B66] Lane D. P. (1992). Cancer. p53, guardian of the genome. Nature 358, 15–16. 10.1038/358015a0 1614522

[B67] Latchman D. S. (2008). Eukaryotic Transcription Factors. New York: Academic Press.

[B68] Lin C.-Y. Navarro S. Reddy S. Comai L. (2006). CK2-mediated stimulation of Pol I transcription by stabilization of UBF-SL1 interaction. Nucleic Acids Res. 34, 4752–4766. 10.1093/nar/gkl581 16971462 PMC1635259

[B69] Lin Q. Luo X. Sawadogo M. (1994). Archaic structure of the gene encoding transcription factor USF. J. Biol. Chem. 269, 23894–23903. 7523363

[B70] Liu C. Sista N. D. Pagano J. S. (1996). Activation of the Epstein-Barr virus DNA polymerase promoter by the BRLF1 immediate-early protein is mediated through USF and E2F. J. Virol. 70, 2545–2555. 8642684 10.1128/jvi.70.4.2545-2555.1996 PMC190100

[B71] Löffler I. Grün M. Böhmer F. D. Rubio I. (2008). Role of cAMP in the promotion of colorectal cancer cell growth by Prostaglandin E2. BMC Cancer 8:380. 10.1186/1471-2407-8-380 19099561 PMC2615781

[B72] Luo J. (2009). Glycogen synthase kinase 3β (GSK3β) in tumorigenesis and cancer chemotherapy. Cancer Lett. 273, 194–200. 10.1016/j.canlet.2008.05.045 18606491 PMC4978950

[B73] Luo X. Sawadogo M. (1996a). Antiproliferative properties of the USF family of helix-loop-helix transcription factors. Proc. Natl. Acad. Sci. U.S.A. 93, 1308–1313. 10.1073/pnas.93.3.1308 8577760 PMC40076

[B74] Luo X. Sawadogo M. (1996b). Functional domains of the transcription factor USF2: atypical nuclear localization signals and context-dependent transcriptional activation domains. Mol. Cell. Biol. 16, 1367–1375. 8657110 10.1128/mcb.16.4.1367 PMC231121

[B75] Lupp S. Götz C. Khadouma S. Horbach T. Dimova E. Y. Bohrer A. M. (2014). The upstream stimulatory factor USF1 is regulated by protein kinase CK2 phosphorylation. Cell. Signal. 26, 2809–2817. 10.1016/j.cellsig.2014.08.028 25194820

[B76] Maekawa T. Sudo T. Kurimoto M. Ishii S. (1991). USF-related transcription factor, HIV-TF1, stimulates transcription of human immunodeficiency virus-1. Nucleic Acids Res. 19, 4689–4694. 10.1093/nar/19.17.4689 1653950 PMC328710

[B77] Meier J. L. Straus S. E. (1995). Interactions between varicella-zoster virus IE62 and cellular transcription factor USF in the coordinate activation of genes 28 and 29. Neurology 45, S30–S32 10.1212/WNL.45.12_Suppl_8.S30 8545014

[B78] Miyamoto N. G. Moncollin V. Egly J. M. Chambon P. (1985). Specific interaction between a transcription factor and the upstream element of the adenovirus-2 major late promoter. EMBO J. 4, 3563–3570. 4092688 10.1002/j.1460-2075.1985.tb04118.x PMC554698

[B79] Montenarh M. (2010). Cellular regulators of protein kinase CK2. Cell Tissue Res. 342, 139–146. 10.1007/s00441-010-1068-3 20976471

[B80] Mottet D. Ruys S. P. D. Demazy C. Raes M. Michiels C. (2005). Role for casein kinase 2 in the regulation of HIF-1 activity. Int. J. Cancer 117, 764–774. 10.1002/ijc.21268 15957168

[B81] Nowak M. Helleboid-Chapman A. Jakel H. Martin G. Duran-Sandoval D. Staels B. (2005). Insulin-mediated down-regulation of apolipoprotein A5 gene expression through the phosphatidylinositol 3-kinase pathway: role of upstream stimulatory factor. Mol. Cell. Biol. 25, 1537–1548. 10.1128/MCB.25.4.1537-1548.2005 15684402 PMC548024

[B82] Nupponen N. N. Hyytinen E. R. Kallioniemi A. H. Visakorpi T. (1998). Genetic alterations in prostate cancer cell lines detected by comparative genomic hybridization. Cancer Genet. Cytogenet. 101, 53–57 10.1016/S0165-4608(97)00060-5 9460501

[B83] Ocejo-Garcia M. Baokbah T. A. Ashurst H. L. Cowlishaw D. Soomro I. Coulson J. M. (2005). Roles for USF-2 in lung cancer proliferation and bronchial carcinogenesis. J. Pathol. 206, 151–159. 10.1002/path.1775 15856526

[B84] Olave N. C. Grenett M. H. Cadeiras M. Grenett H. E. Higgins P. J. (2010). Upstream stimulatory factor-2 mediates quercetin-induced suppression of PAI-1 gene expression in human endothelial cells. J. Cell. Biochem. 111, 720–726. 10.1002/jcb.22760 20626032 PMC3521593

[B85] Park B. J. Park J. I. Byun D. S. Park J. H. Chi S. G. (2000). Mitogenic conversion of transforming growth factor-β1 effect by oncogenic Ha-Ras-induced activation of the mitogen-activated protein kinase signaling pathway in human prostate cancer. Cancer Res. 60, 3031–3038. 10850453

[B86] Pawlus M. R. Wang L. Ware K. Hu C. J. (2012). Upstream stimulatory factor 2 and hypoxia-inducible factor 2α (HIF2α) cooperatively activate HIF2 target genes during hypoxia. Mol. Cell. Biol. 32, 4595–4610. 10.1128/MCB.00724-12 22966206 PMC3486188

[B87] Pognonec P. Boulukos K. E. Aperlo C. Fujimoto M. Ariga H. Nomoto A. (1997). Cross-family interaction between the bHLHZip USF and bZip Fra1 proteins results in down-regulation of AP1 activity. Oncogene 14, 2091–2098. 10.1038/sj.onc.1201046 9160889

[B88] Porter S. E. Dwyer-Nield L. D. Malkinson A. M. (2001). Regulation of lung epithelial cell morphology by cAMP-dependent protein kinase type I isozyme. Am. J. Physiol. 280, L1282–L1289. 10.1152/ajplung.2001.280.6.L1282 11350809

[B89] Providence K. M. White L. A. Tang J. Gonclaves J. Staiano-Coico L. Higgins P. J. (2002). Epithelial monolayer wounding stimulates binding of USF-1 to an E-box motif in the plasminogen activator inhibitor type 1 gene. J. Cell. Sci. 115, 3767–3777. 10.1242/jcs.00051 12235287

[B90] Qi L. Allen R. R. Lu Q. Higgins C. E. Garone R. Staiano-Coico L. (2006). PAI-1 transcriptional regulation during the G0 → G1 transition in human epidermal keratinocytes. J. Cell. Biochem. 99, 495–507. 10.1002/jcb.20885 16622840

[B91] Qyang Y. Luo X. Lu T. Ismail P. M. Krylov D. Vinson C. (1999). Cell-type-dependent activity of the ubiquitous transcription factor USF in cellular proliferation and transcriptional activation. Mol. Cell. Biol. 19, 1508–1517. 9891084 10.1128/mcb.19.2.1508 PMC116079

[B92] Rada-Iglesias A. Ameur A. Kapranov P. Enroth S. Komorowski J. Gingeras T. R. (2008). Whole-genome maps of USF1 and USF2 binding and histone H3 acetylation reveal new aspects of promoter structure and candidate genes for common human disorders. Genome Res. 18, 380–392. 10.1101/gr.6880908 18230803 PMC2259102

[B93] Reisman D. Rotter V. (1993). The helix-loop-helix containing transcription factor USF binds to and transactivates the promoter of the p53 tumor suppressor gene. Nucleic Acids Res. 21, 345–350. 10.1093/nar/21.2.345 8441640 PMC309112

[B94] Riccio A. Pedone P. V. Lund L. R. Olesen T. Olsen H. S. Andreasen P. A. (1992). Transforming growth factor beta 1-responsive element: closely associated binding sites for USF and CCAAT-binding transcription factor-nuclear factor I in the type 1 plasminogen activator inhibitor gene. Mol. Cell. Biol. 12, 1846–1855. 1549130 10.1128/mcb.12.4.1846-1855.1992 PMC369628

[B95] Rodriguez C. I. Girones N. Fresno M. (2003). Cha, a basic helix-loop-helix transcription factor involved in the regulation of upstream stimulatory factor activity. J. Biol. Chem. 278, 43135–43145. 10.1074/jbc.M300053200 12923186

[B96] Roy A. L. Du H. Gregor P. D. Novina C. D. Martinez E. Roeder R. G. (1997). Cloning of an inr- and E-box-binding protein, TFII-I, that interacts physically and functionally with USF1. EMBO J. 16, 7091–7104. 10.1093/emboj/16.23.7091 9384587 PMC1170311

[B97] Sabio G. Davis R. J. (2014). TNF and MAP kinase signalling pathways. Semin. Immunol. 26, 237–245. 10.1016/j.smim.2014.02.009 24647229 PMC4099309

[B98] Saito T. Oishi T. Yanai K. Shimamoto Y. Fukamizu A. (2003). Cloning and characterization of a novel splicing isoform of USF1. Int. J. Mol. Med. 12, 161–167. 10.3892/ijmm.12.2.161 12851711

[B99] Samarakoon R. Higgins C. E. Higgins S. P. Higgins P. J. (2009). TGF-β1-induced expression of the poor prognosis SERPINE1/PAI-1 gene requires EGFR signaling: a new target for anti-EGFR therapy. J. Oncol. 2009, 342391. 10.1155/2009/342391 19365582 PMC2667932

[B100] Samoylenko A. Dimova E. Y. Horbach T. Teplyuk N. Immenschuh S. Kietzmann T. (2008). Opposite expression of the antioxidant heme oxygenase-1 in primary cells and tumor cells: regulation by interaction of USF-2 and Fra-1. Antioxid. Redox Signal. 10, 1163–1174. 10.1089/ars.2007.1968 18331200

[B101] Samoylenko A. Roth U. Jungermann K. Kietzmann T. (2001). The upstream stimulatory factor-2a inhibits plasminogen activator inhibitor-1 gene expression by binding to a promoter element adjacent to the hypoxia-inducible factor-1 binding site. Blood 97, 2657–2666. 10.1182/blood.V97.9.2657 11313255

[B102] Satyanarayana A. Kaldis P. (2009). Mammalian cell-cycle regulation: several Cdks, numerous cyclins and diverse compensatory mechanisms. Oncogene 28, 2925–2939. 10.1038/onc.2009.170 19561645

[B103] Sawadogo M. (1988). Multiple forms of the human gene-specific transcription factor USF. II. DNA binding properties and transcriptional activity of the purified HeLa USF. J. Biol. Chem. 263, 11994–12001. 3403559

[B104] Sawadogo M. Roeder R. G. (1985). Interaction of a gene-specific transcription factor with the adenovirus major late promoter upstream of the TATA box region. Cell 43, 165–175 10.1016/0092-8674(85)90021-2 4075392

[B105] Sayasith K. Lussier J. G. Sirois J. (2005). Role of upstream stimulatory factor phosphorylation in the regulation of the prostaglandin G/H synthase-2 promoter in granulosa cells. J. Biol. Chem. 280, 28885–28893. 10.1074/jbc.M413434200 15927963

[B106] Schneider C. C. Ampofo E. Montenarh M. (2012). CK2 regulates ATF4 and CHOP transcription within the cellular stress response signalling pathway. Cell. Signal. 24, 1797–1802. 10.1016/j.cellsig.2012.05.006 22609407

[B107] Seufferlein T. Rozengurt E. (1995). Sphingosylphosphorylcholine rapidly induces tyrosine phosphorylation of p125^FAK^ and paxillin, rearrangement of the actin cytoskeleton and focal contact assembly. Requirement of p21^rho^ in the signaling pathway. J. Biol. Chem. 270, 24343–24351. 10.1074/jbc.270.41.24343 7592646

[B108] Shi L. Liu S. Nikolic D. Wang S. (2008). High glucose levels upregulate upstream stimulatory factor 2 gene transcription in mesangial cells. J. Cell. Biochem. 103, 1952–1961. 10.1002/jcb.21585 17955499 PMC9084927

[B109] Shieh B. H. Sparkes R. S. Gaynor R. B. Lusis A. J. (1993). Localization of the gene-encoding upstream stimulatory factor (USF) to human chromosome 1q22-q23. Genomics 16, 266–268. 10.1006/geno.1993.1174 8486371

[B110] Sieweke M. H. Tekotte H. Jarosch U. Graf T. (1998). Cooperative interaction of ets-1 with USF-1 required for HIV-1 enhancer activity in T cells. EMBO J. 17, 1728–1739. 10.1093/emboj/17.6.1728 9501094 PMC1170520

[B111] Sirito M. Lin Q. Deng J. M. Behringer R. R. Sawadogo M. (1998). Overlapping roles and asymmetrical cross-regulation of the USF proteins in mice. Proc. Natl. Acad. Sci. U.S.A. 95, 3758–3763. 10.1073/pnas.95.7.3758 9520440 PMC19910

[B112] Sirito M. Lin Q. Maity T. Sawadogo M. (1994). Ubiquitous expression of the 43-and 44-kDa forms of transcription factor USF in mammalian cells. Nucleic Acids Res. 22, 427–433. 10.1093/nar/22.3.427 8127680 PMC523599

[B113] Sirito M. Walker S. Lin Q. Kozlowski M. T. Klein W. H. Sawadogo M. (1992). Members of the USF family of helix-loop-helix proteins bind DNA as homo- as well as heterodimers. Gene Expr. 2, 231–240. 1450663 PMC6057381

[B114] Steingrimsson E. Sawadogo M. Gilbert D. J. Zervos A. S. Brent R. Blanar M. A. (1995). Murine chromosomal location of five bHLH-Zip transcription factor genes. Genomics 28, 179–183. 10.1006/geno.1995.1129 8530024

[B115] Stratakis C. A. (2013). cAMP/PKA signaling defects in tumors: genetics and tissue-specific pluripotential cell-derived lesions in human and mouse. Mol. Cell. Endocrinol. 371, 208–220. 10.1016/j.mce.2013.01.015 23485729 PMC3625474

[B116] Terragni J. Nayak G. Banerjee S. Medrano J. L. Graham J. R. Brennan J. F. (2011). The E-Box binding factors Max/Mnt, MITF and USF1 Act coordinately with FoxO to regulate expression of Pro-apoptotic and cell cycle control genes by phosphatidylinositol 3-kinase/Akt/GSK3 signaling. J. Biol. Chem. 286, 36215–36227. 10.1074/jbc.M111.246116 21873430 PMC3196102

[B117] Tiruppathi C. Soni D. Wang D. M. Xue J. Singh V. Thippegowda P. B. (2014). The transcription factor DREAM represses the deubiquitinase A20 and mediates inflammation. Nat. Immunol. 15, 239–247. 10.1038/ni.2823 24487321 PMC4005385

[B118] Vallet V. S. Casado M. Henrion A. A. Bucchini D. Raymondjean M. Kahn A. (1998). Differential roles of upstream stimulatory factors 1 and 2 in the transcriptional response of liver genes to glucose. J. Biol. Chem. 273, 20175–20179. 10.1074/jbc.273.32.20175 9685363

[B119] Vallet V. S. Henrion A. A. Bucchini D. Casado M. Raymondjean M. Kahn A. (1997). Glucose-dependent liver gene expression in upstream stimulatory factor 2–/– mice. J. Biol. Chem. 272, 21944–21949. 10.1074/jbc.272.35.21944 9268329

[B120] Van de Sande T. De Schrijver E. Heyns W. Verhoeven G. Swinnen J. V. (2002). Role of the phosphatidylinositol 3′-kinase/PTEN/Akt kinase pathway in the overexpression of fatty acid synthase in LNCaP prostate cancer cells. Cancer Res. 62, 642–646. 11830512

[B121] Verger A. Perdomo J. Crossley M. (2003). Modification with SUMO. EMBO Rep. 4, 137–142. 10.1038/sj.embor.embor738 12612601 PMC1315836

[B122] Viart V. Varilh J. Lopez E. Rene C. Claustres M. Taulan-Cadars M. (2013). Phosphorylated C/EBPβ influences a complex network involving YY1 and USF2 in lung epithelial cells. PLoS ONE 8:e60211. 10.1371/journal.pone.0060211 23560079 PMC3613372

[B123] Viollet B. Lefrancois-Martinez A. M. Henrion A. Kahn A. Raymondjean M. Martinez A. (1996). Immunochemical characterization and transacting properties of upstream stimulatory factor isoforms. J. Biol. Chem. 271, 1405–1415. 10.1074/jbc.271.3.1405 8576131

[B124] Welker S. Götz C. Servas C. Laschke M. W. Menger M. D. Montenarh M. (2013). Glucose regulates protein kinase CK2 in pancreatic β-cells and its interaction with PDX-1. Int. J. Biochem. Cell Biol. 45, 2786–2795. 10.1016/j.biocel.2013.10.002 24126110

[B125] White L. A. Bruzdzinski C. Kutz S. M. Gelehrter T. D. Higgins P. J. (2000). Growth state-dependent binding of USF-1 to a proximal promoter E box element in the rat plasminogen activator inhibitor type 1 gene. Exp. Cell Res. 260, 127–135. 10.1006/excr.2000.5001 11010817

[B126] Whitmarsh A. J. Davis R. J. (2007). Role of mitogen-activated protein kinase kinase 4 in cancer. Oncogene 26, 3172–3184. 10.1038/sj.onc.1210410 17496914

[B127] Wierstra I. (2011). The transcription factor FOXM1c is activated by protein kinase CK2, protein kinase A (PKA), c-Src and Raf-1. Biochem. Biophys. Res. Commun. 413, 230–235. 10.1016/j.bbrc.2011.08.075 21875579

[B128] Wong R. H. Sul H. S. (2009). DNA-PK: relaying the insulin signal to USF in lipogenesis. Cell Cycle 8, 1977–1978. 10.4161/cc.8.13.8941 19550139 PMC2862631

[B129] Woodgett J. R. (1990). Molecular cloning and expression of glycogen synthase kinase-3/factor A. EMBO J. 9, 2431–2438. 2164470 10.1002/j.1460-2075.1990.tb07419.x PMC552268

[B130] Wu K. Jiang S. W. Couch F. J. (2003). p53 mediates repression of the BRCA2 promoter and down-regulation of BRCA2 mRNA and protein levels in response to DNA damage. J. Biol. Chem. 278, 15652–15660. 10.1074/jbc.M211297200 12591928

[B131] Wutthisathapornchai A. Vongpipatana T. Muangsawat S. Boonsaen T. MacDonald M. J. Jitrapakdee S. (2014). Multiple e-boxes in the distal promoter of the rat pyruvate carboxylase gene function as a glucose-responsive element. PLoS ONE 9:e102730. 10.1371/journal.pone.0102730 25054881 PMC4108332

[B132] Xiao Q. Kenessey A. Ojamaa K. (2002). Role of USF1 phosphorylation on cardiac α-myosin heavy chain promoter activity. Am. J. Physiol. 283, H213–H219 10.1152/ajpheart.01085.2001 12063293

[B133] Yang Y. A. Han W. F. Morin P. J. Chrest F. J. Pizer E. S. (2002). Activation of fatty acid synthesis during neoplastic transformation: role of mitogen-activated protein kinase and phosphatidylinositol 3-kinase. Exp. Cell Res. 279, 80–90. 10.1006/excr.2002.5600 12213216

