# Corrigendum: Protein kinases as switches for the function of upstream stimulatory factors: implications for tissue injury and cancer

**DOI:** 10.3389/fphar.2016.00092

**Published:** 2016-04-05

**Authors:** Tina Horbach, Claudia Götz, Thomas Kietzmann, Elitsa Y. Dimova

**Affiliations:** ^1^Faculty of Biochemistry and Molecular Medicine, Biocenter Oulu, University of OuluOulu, Finland; ^2^Department of Chemistry, University of KaiserslauternKaiserslautern, Germany; ^3^Medical Biochemistry and Molecular Biology, Saarland UniversityHomburg, Germany

**Keywords:** USF, phosphorylation, transcription factor, cancer, tumor, signaling, therapeutic target

Correct an error in the reference list. The correction does not affect the scientific validity of the results.

Reference 14 in our paper is wrong:

Refrence 14: Bussiere M., Vance J. E., Campenot R. B., Vance D. E. (2001). Compartmentalization of choline and acetylcholine metabolism in cultured sympathetic neurons. J. Biochem. (Tokyo) 130, 561–568 10.1093/oxfordjournals.jbchem.a003019 is not correct

Correct one citation is:

Bussière, F. I., Michel, V., Mémet, S., Avé, P., Vivas, J. R., Huerre, M., et al. (2010). H. pylori-induced promoter hypermethylation downregulates USF1 and USF2 transcription factor gene expression. *Cell Microbiol.* 12, 1124–1133. doi: 10.1111/j.1462-5822.2010.01457.x

## Author contributions

All authors listed, have made substantial, direct and intellectual contribution to the work, and approved it for publication.

### Conflict of interest statement

The authors declare that the research was conducted in the absence of any commercial or financial relationships that could be construed as a potential conflict of interest.

